# Supplementary material for: Single-cell transcriptomic analysis highlights origin and pathological process of human endometrioid endometrial carcinoma
Source: Nat Commun. 2022 Oct 22;13:6300. doi: 10.1038/s41467-022-33982-7 (PMC9588071; doi:10.1038/s41467-022-33982-7)
Supplement: Supplementary file 1 — Supplementary Information [file 41467_2022_33982_MOESM1_ESM.pdf]

Supplementary Information

Single-Cell Transcriptomic Analysis Highlights Origin and Pathological Process of Human Endometrioid Endometrial Carcinoma

Ren *et al*.

Supplemental Figures S1-S8

Supplemental Table S1-S2

Figure S1

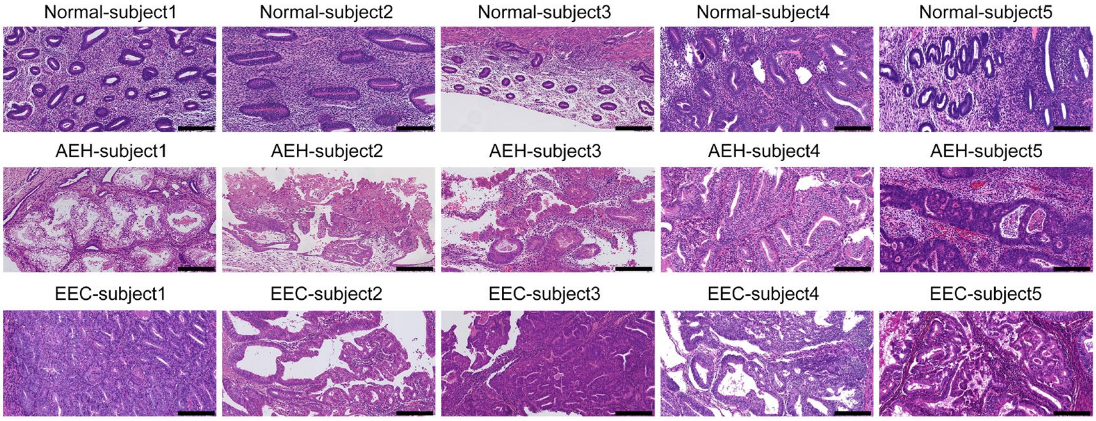

Figure S1. Representative images of H&E staining of indicated endometrial tissues enrolled in the study. Normal: normal endometrium; AEH: atypical endometrial hyperplasia; EEC: endometrioid endometrial cancer. The scale bar is 200 μm.

Figure S2

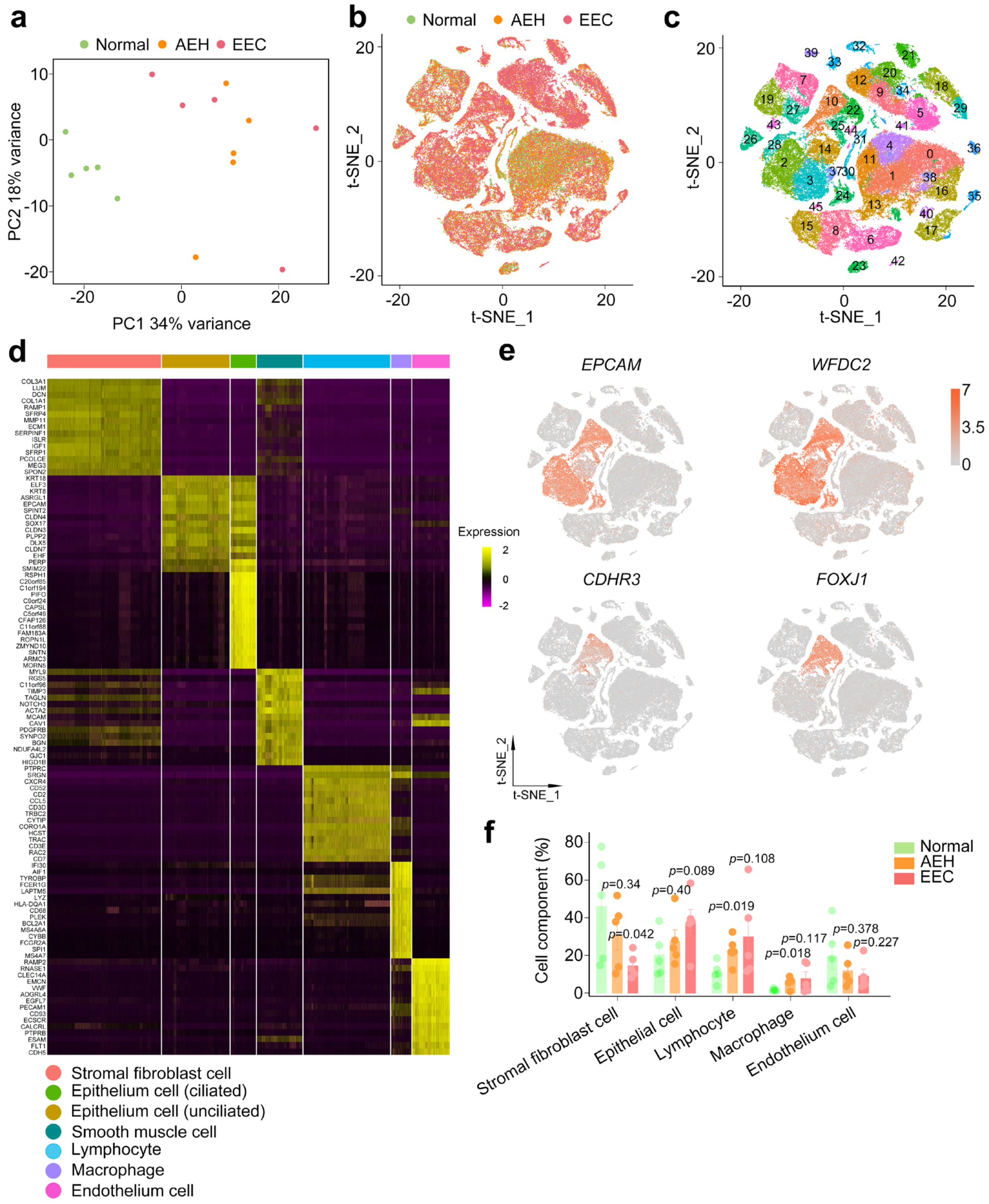

Figure S2. Overview of normal endometrium, AEH tissue and EEC tissue via single-cell sequencing. (a) PCA analysis showed transcriptome relationship among Normal, AEH and EEC samples. **(b, c)** t-SNE of the 99,215 cells profiled here, with each cell color coded for: its sample type (Normal, AEH and EEC) (b) and its cell cluster (0 to 45) (c). **(d)** Heatmap of top 15 marker genes of different cell types. **(e)** Expression pattern of epithelium (*EPCAM* and *WFDC2*) and ciliated epithelium (*CDHR3* and *FOXJ1*) marker genes. **(f)** The percentage of different cell types at different stages of endometrial pathology. Data are mean ± SEM based on five independent biological replicates. Significance was evaluated by comparing with normal group (the significance is analyzed by *t*-test, two-sided, and *P-values* are denoted in the picture above).

Figure S3

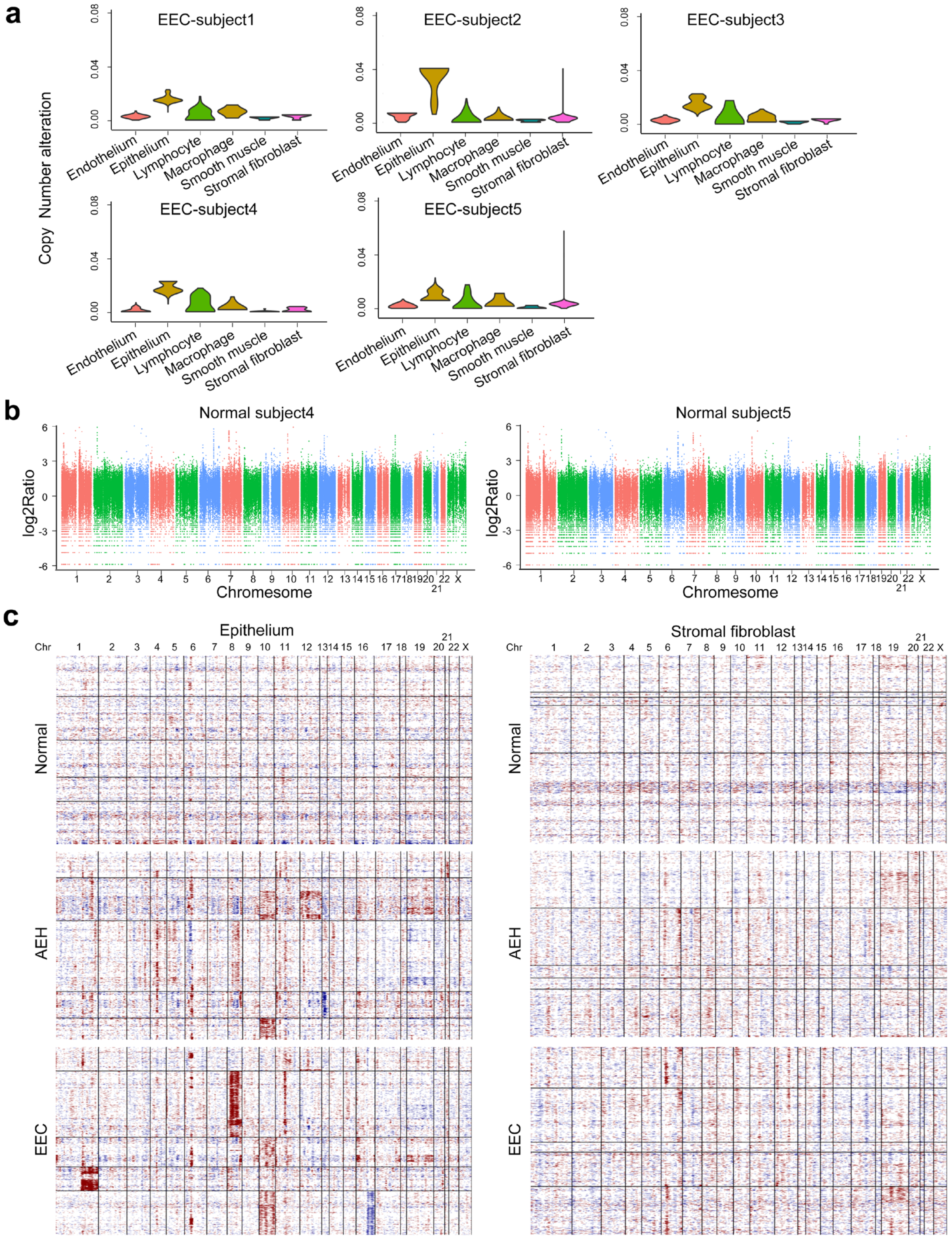

Figure S3. Copy number variation analysis via the whole genome sequencing (WES) and inferred CNV. **(a)** Copy number variation score of EEC subjects case by case. According to the molecular subtypes, EEC-subject 1, 2 and 3 are classified into NSMP (no specific molecular profile), whereas EEC-subject 4 and 5 are classified into MMRd (mismatch repair deficient). **(b)** WES was performed on two normal endometrium (normal subject 4 and normal subject5), and there is no obvious CNV. **(c)** Copy number variation analysis was applied in epithelium (left) and stromal fibroblast cells (right) of five EEC independent samples.

Figure S4

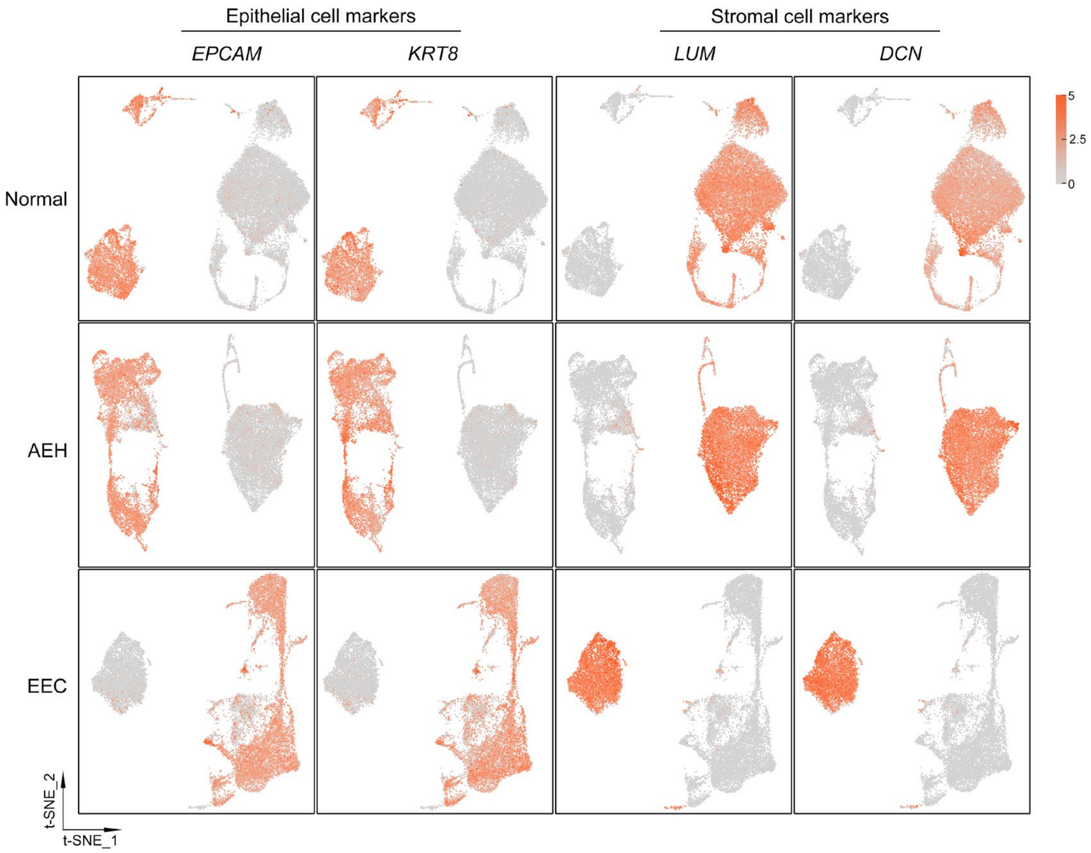

Figure S4. Marker expression pattern of epithelium and stromal fibroblast in normal, AEH and EEC samples. *EPCAM* and *KRT5* are epithelium markers; *LUM* and *DCN* are stromal fibroblast markers.

Figure S5

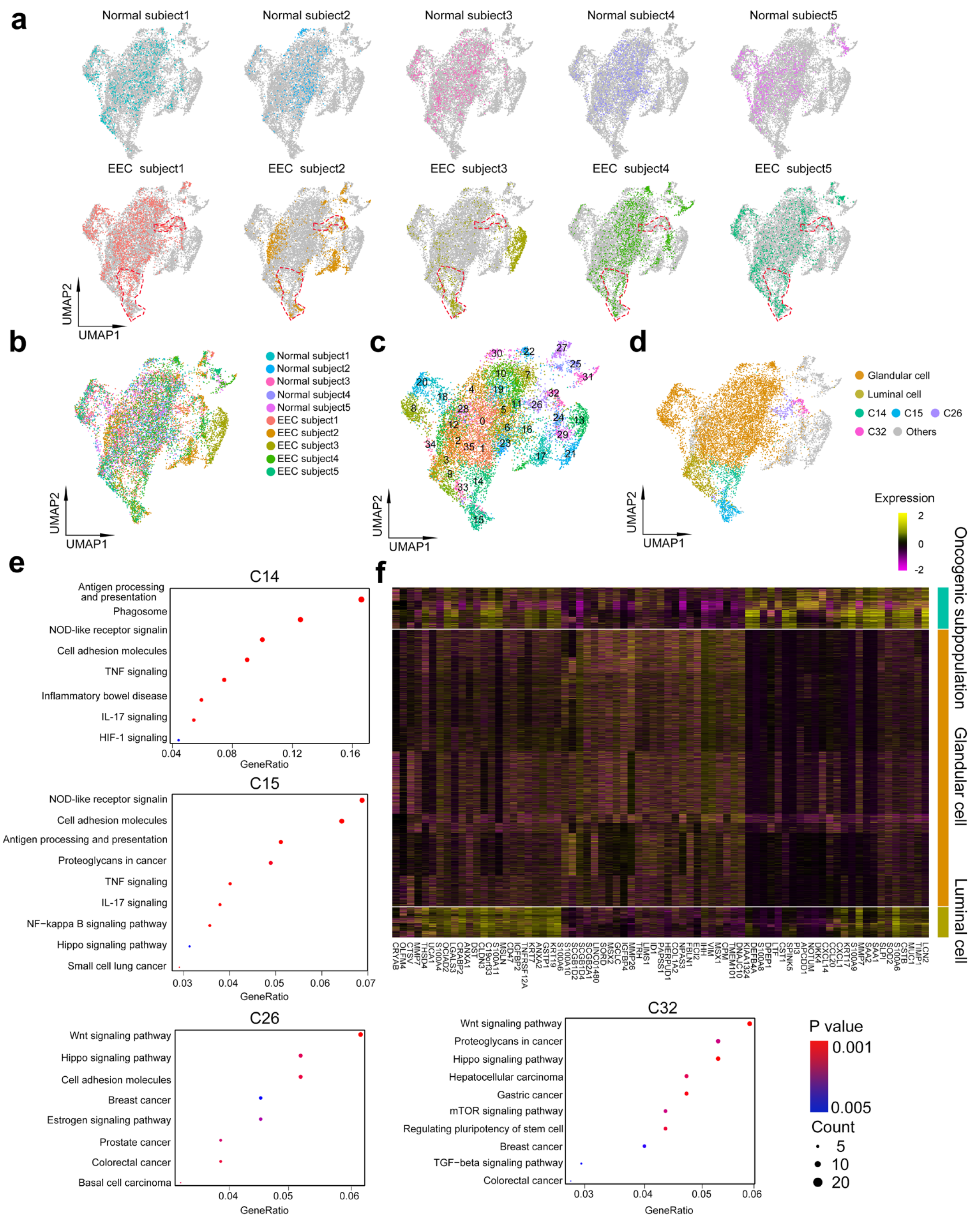

Figure S5. Characterization of unciliated epithelium in normal and EEC samples. (a, b) t-SNE plot of unciliated epithelium from normal and EEC samples, color-coded by their patient ID. Oncogenic subpopulation in each EEC sample were circled. **(c, d)** t-SNE plot of unciliated epithelium from normal and EEC samples grouped by unsupervised clustering (c), and manually annotation (d). **(e)** KEGG enrichment analysis showed associated signaling pathways of oncogenic subpopulation 14,15,26 and 32. Data are analyzed by Fisher's exact test, one-sided (Source data are provided as a Source Data file). **(f)** Heatmap of the expression of top 25 marker genes of EEC oncogenic subpopulation, glandular and luminal cells.

Figure S6

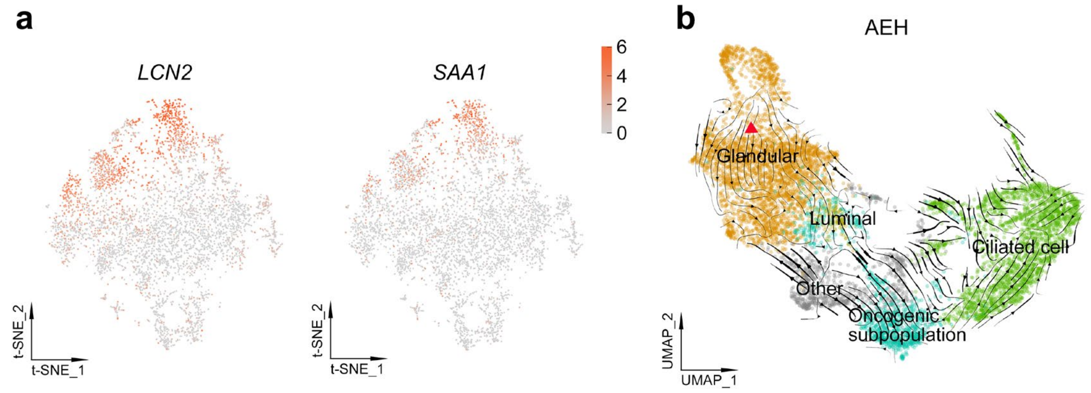

Figure S6. Signature genes pattern and RNA-velocity map of epithelial cells in AEH. (a) Expression pattern of *LCN2* and *SAA1* of epithelial cells in AEH. **(b)** RNA velocity map of epithelial cells of AEH samples. Arrows indicate the flow direction and the red triangle indicates the initial position of RNA stream.

Figure S7

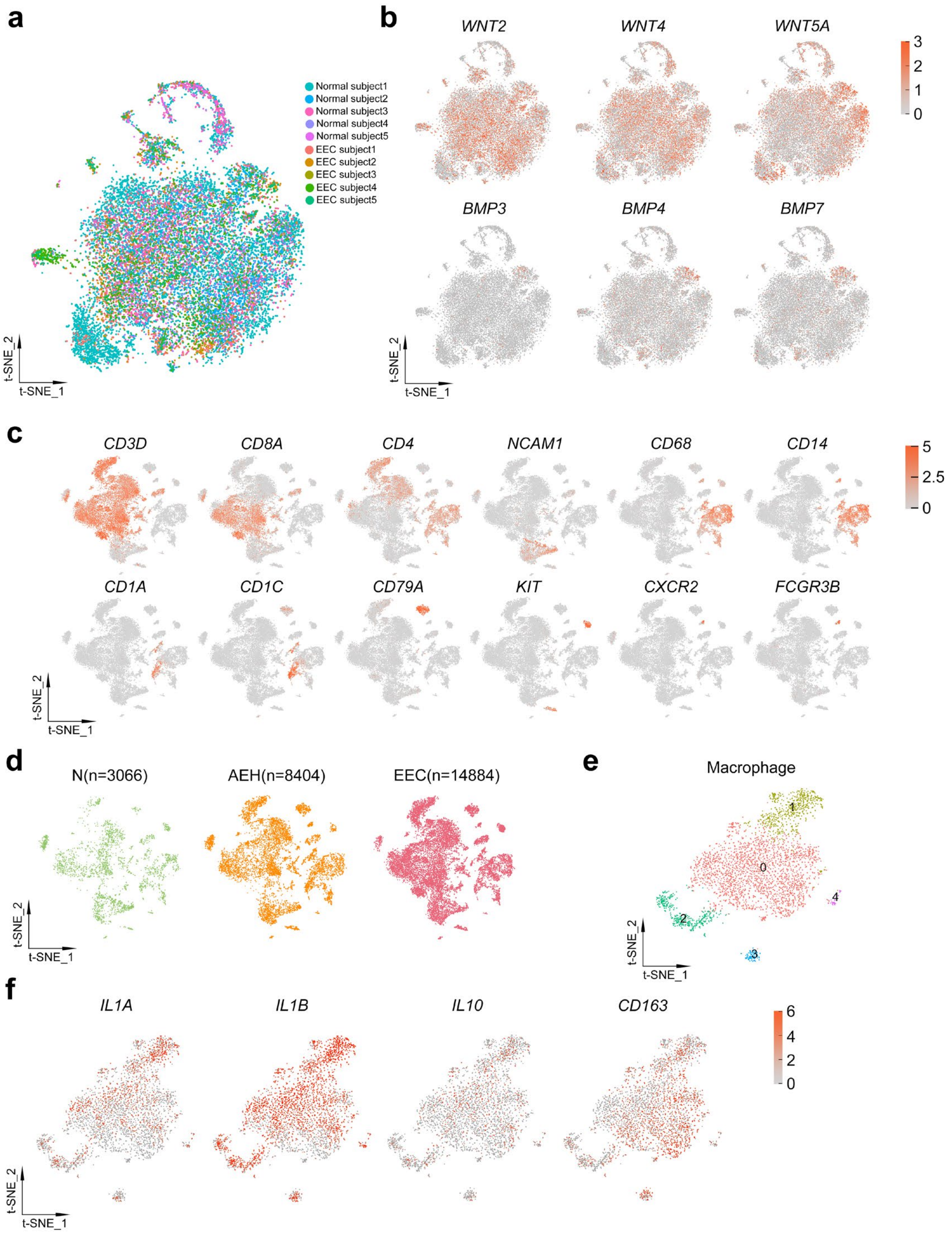

Figure S7. Characterization of microenvironment of normal, AEH and EEC samples. (a) t-SNE plot of stromal fibroblast from normal and EEC samples, color-coded by their patient ID. **(b)** Expression pattern of representative genes in BMP+ fibroblast (positive for *BMP3*, *BMP4* and *BMP7*) and WNT niche related fibroblast (*WNT2*, *WNT4* and *WNT5A*). **(c)** Expression pattern of canonical marker genes in different immune subtypes. **(d)** Distribution pattern of immune cells in normal, AEH and EEC samples, respectively. **(e)** t-SNE plot of macrophage, grouped by unsupervised clustering (clusters 0-4). **(f)** Expression pattern of canonical M1-type (*IL1A* and *IL1B*)

and M2-type (*IL10* and *CD163*) marker genes in macrophage.

Figure S8

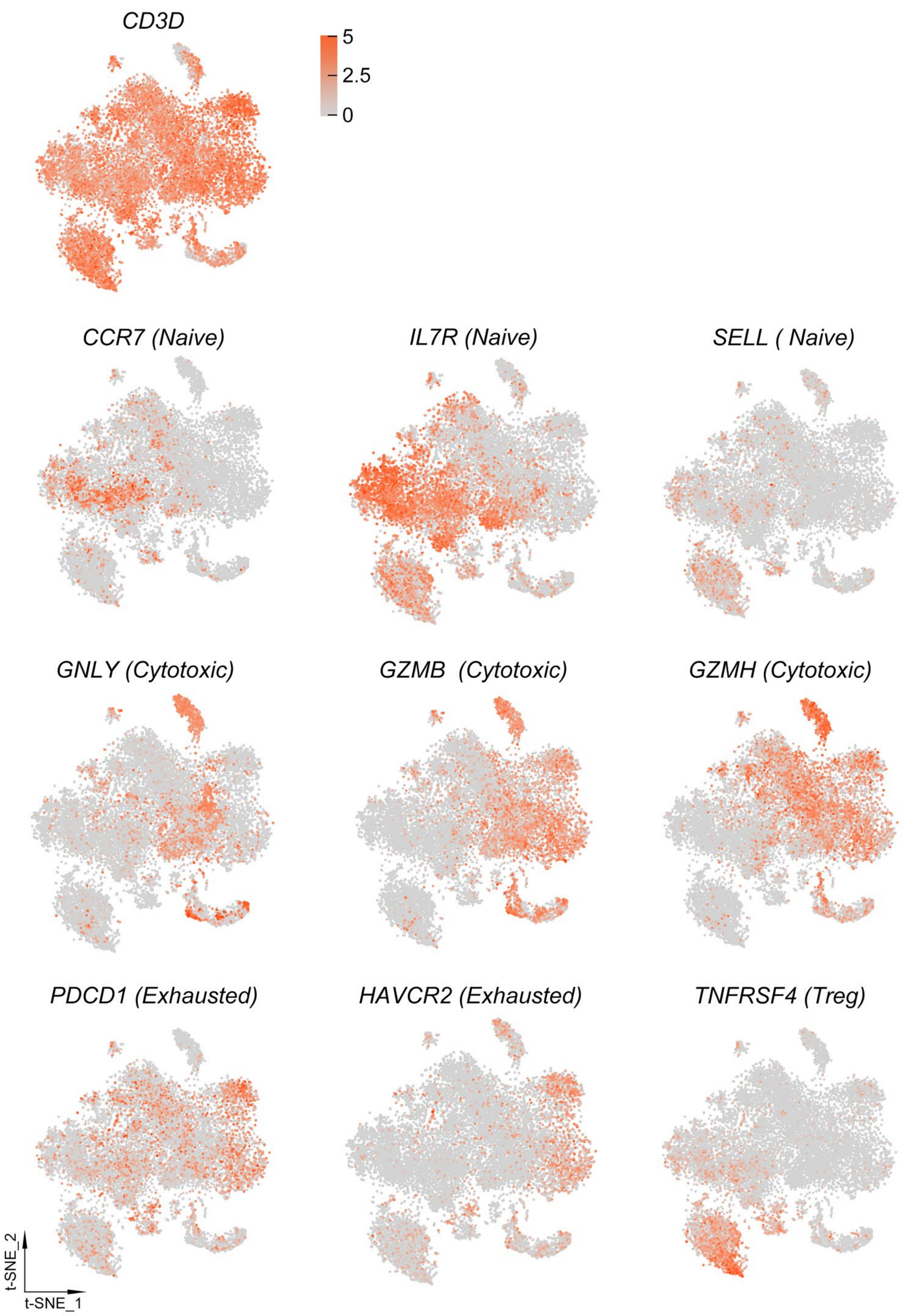

Figure S8. Expression patterns of classical marker genes in different states in T cells. T cells are classified into naïve, cytotoxic, exhausted and Treg states, and the corresponding markers are denoted above.

Table S1. The clinical information of the donors enrolled in the study.

Patient ID	Age (y)	Parity	BMI (Kg/m^2)	Identification for the enrollment	Gynecological diseases and other complications	Molecular subtypes
Normal -subjuect1	47	0	26.3	Normal endometrium with proliferative changes	Hyperthyroidism; adenomyosis	
Normal -subjuect2	47	1	22.4	Normal endometrium with proliferative changes	Cervical cancer	
Normal -subjuect3	52	1	23.1	Normal endometrium with proliferative changes	Adenomyosis; uterine fibroids	
Normal -subject4	50	1	25.6	Normal endometrium with proliferative changes	Uterine fibroids	
Normal -subject5	51	3	25.7	Normal endometrium with proliferative changes	Cervical cancer	
AEH-subject1	49	4	23.8	AEH	Uterine fibroids	
AEH-subject2	54	2	23.4	AEH	Hypertension; impaired glucose tolerance	
AEH-subject3	48	1	25.3	AEH	Anemia; hyperthyroidism	
AEH-subject4	49	1	29.2	AEH	Uterine fibroids	
AEH-subject5	46	1	23.8	AEH	Uterine fibroids; adenomyosis	
EEC-subject1	50	1	37.4	EEC, grade 1	Hypertension; uterine fibroids	NSMP
EEC-subject2	51	1	25.7	EEC, grade 1	Hypertension; impaired glucose tolerance; adenomyosis	NSMP
EEC-subject3	44	1	32.9	EEC, grade 1	Hypertension	NSMP
EEC-subject4	49	2	26.3	EEC, grade 1	Diabetes (type II); uterine fibroids	MMRd
EEC-subject5	52	1	29.8	EEC, grade 1	Impaired glucose tolerance	MMRd
NF-subject1	47	1	26.0	Normal endometrium With mid-secretory endometrium	Uterine fibroids	
NF-subject2	49	2	23.0	Normal endometrium With mid-secretory endometrium	Uterine fibroids	
CAF-subject1	57	1	19.1	EEC, grade 2	Uterine fibroids; adenomyosis	
CAF-subject2	55	1	28.1	EEC, grade 1	Hypertension; uterine fibroids	

Notes: "y" denotes "years old"; "Normal" denotes "Normal endometrium"; "AEH" denotes "Endometrial atypical hyperplasia"; "EEC" denotes "Endometrioid endometrial cancer"; "NF" denotes "Normal endometrial fibroblasts"; "CAF" denotes "Cancer associated fibroblasts"; "NSMP" denotes "no specific molecular profile"; "MMRd" denotes "Mismatch repair deficient".

Table S2. The single-cell RNA-seq dataset of the donors enrolled in the study.

Patient ID	Estimated Number of Cells	Cell Number After Quality Filter	Median Genes Per Cell (Range)	Median UMI Counts Per Cell	Mean reads Per Cell	Reads Mapped Confidently to Genome	Reads Mapped Confidently to Transcriptome
Normal -subjuect1	10,845	10,474	2,540 (307~7,736)	6,980	33,029	92.3%	55.1%
Normal -subjuect2	8,013	7,147	2,582 (353~8,198)	7,152	37,910	94.8%	55.1%
Normal -subjuect3	8,242	6,996	2,504 (326~8,892)	7,452	42,534	92.0%	55.7%
Normal -subjuect4	6,346	4,991	2,700 (320~8,416)	7,766	60,268	96.1%	55.7%
Normal -subjuect5	4,567	3,097	2,912 (298~9,285)	7,998	67,717	95.0%	49.0%
AEH-subject1	6,159	5,517	1,587 (329~8,602)	4,143	49,714	94.6%	58.6%
AEH-subject2	9,852	9,190	2,138 (149~8,262)	6,258	44,016	94.6%	56.2%
AEH-subject3	7,726	7,170	2,734 (316~7,626)	8,766	45,031	92.6%	63.3%
AEH-subject4	7,431	6,356	2,200 (215~8,233)	5,973	46,266	93.9%	65.0%
AEH-subject5	8,436	6,586	2,084 (348~8,905)	5,922	38,558	96.4%	59.8%
EEC-subject1	11,868	9,749	1,322 (255~7,332)	3,652	28,134	94.9%	59.7%
EEC-subject2	8,810	6,840	1,279 (321~7,794)	3,030	33,816	85.8%	52.9%
EEC-subject3	4,816	3,645	1,726 (260~9,222)	6,092	61,572	91.2%	60.2%
EEC-subject4	13,207	11,082	1,718 (278~7,716)	4,420	29,200	95.6%	67.2%
EEC-subject5	12,407	10,174	1,268 (340~7,403)	3,076	23,430	95.7%	64.5%
