# Supplementary material for: Single-cell transcriptomic analysis highlights origin and pathological process of human endometrioid endometrial carcinoma
Source: Nat Commun. 2022 Oct 22;13:6300. doi: 10.1038/s41467-022-33982-7 (PMC9588071; doi:10.1038/s41467-022-33982-7)
Supplement: Supplementary file 3 — Reporting Summary [file 41467_2022_33982_MOESM3_ESM.pdf]

nature portfolio

Corresponding author(s): Bing Zhao and Xiaojun Chen

Last updated by author(s): Sep 10, 2022

Reporting Summary

Nature Portfolio wishes to improve the reproducibility of the work that we publish. This form provides structure for consistency and transparency in reporting. For further information on Nature Portfolio policies, see our Editorial Policies and the Editorial Policy Checklist.

Statistics

For all statistical analyses, confirm that the following items are present in the figure legend, table legend, main text, or Methods section.

n/a	Confirmed	
☐	☒	The exact sample size (n) for each experimental group/condition, given as a discrete number and unit of measurement
☐	☒	A statement on whether measurements were taken from distinct samples or whether the same sample was measured repeatedly
☐	☒	The statistical test(s) used AND whether they are one- or two-sided<br>*Only common tests should be described solely by name; describe more complex techniques in the Methods section.*
☒	☐	A description of all covariates tested
☐	☒	A description of any assumptions or corrections, such as tests of normality and adjustment for multiple comparisons
☐	☒	A full description of the statistical parameters including central tendency (e.g. means) or other basic estimates (e.g. regression coefficient) AND variation (e.g. standard deviation) or associated estimates of uncertainty (e.g. confidence intervals)
☐	☒	For null hypothesis testing, the test statistic (e.g. F, t, r) with confidence intervals, effect sizes, degrees of freedom and P value noted<br>*Give P values as exact values whenever suitable.*
☒	☐	For Bayesian analysis, information on the choice of priors and Markov chain Monte Carlo settings
☒	☐	For hierarchical and complex designs, identification of the appropriate level for tests and full reporting of outcomes
☒	☐	Estimates of effect sizes (e.g. Cohen's d, Pearson's r), indicating how they were calculated

Our web collection on statistics for biologists contains articles on many of the points above.

Software and code

Policy information about availability of computer code

Data collection	Cell Ranger (v4.0.0)
Data analysis	Prism (v9.2);R (v4.0.4); packages: Seurat (v4.0.0), clusterProfiler (v3.16.1), infercnv (v1.7.1), DESeq2 (1.26.0), Survival (v3.2-13), TCGAbiolinks (2.16.4), scVelo (v0.2.3), velocyto (v0.17.17)<br>The code is available upon request to bingzhao@fudan.edu.cn.

For manuscripts utilizing custom algorithms or software that are central to the research but not yet described in published literature, software must be made available to editors and reviewers. We strongly encourage code deposition in a community repository (e.g. GitHub). See the Nature Portfolio guidelines for submitting code & software for further information.

Data

Policy information about availability of data

All manuscripts must include a data availability statement. This statement should provide the following information, where applicable:

- Accession codes, unique identifiers, or web links for publicly available datasets
- A description of any restrictions on data availability
- For clinical datasets or third party data, please ensure that the statement adheres to our policy

The single-cell RNA sequencing data reported in this paper has been deposited in NCBI SRA database under the accession number SRP349751 [https://www.ncbi.nlm.nih.gov/sra/?term=SRP349751]. WES sequencing data of two normal subjects reported in this paper has been deposited in NCBI SRA database under the accession number of SRP396178 [https://www.ncbi.nlm.nih.gov/sra/?term=SRP396178]. The raw sequencing data are accessible for non-commercial purposes. TCGA-UCEC datasets (https://portal.gdc.cancer.gov/) were also used in this study. All of the data for this manuscript have been made publicly available.

March 2021

Field-specific reporting

Please select the one below that is the best fit for your research. If you are not sure, read the appropriate sections before making your selection.

☒ Life sciences ☐ Behavioural & social sciences ☐ Ecological, evolutionary & environmental sciences

For a reference copy of the document with all sections, see nature.com/documents/nr-reporting-summary-flat.pdf

Life sciences study design

All studies must disclose on these points even when the disclosure is negative.

Sample size	Sample sizes were illustrated in the corresponding Figure legends. No statistical methods were used to predetermine sample sizes. Theoretically, the larger the sample size, the better. However, due to the enrollment requirement and scientific research funds, we selected 5 cases for different disease courses.
Data exclusions	Data were not excluded in the analysis.
Replication	Due to the limited availability of human endometrial tissues and the shortage of expenditure , ScRNA-seq was performed in five independent subjects for each group. Meanwhile, some of the key discoveries derived from the ScRNA-seq were tested in another independent subjects, and details were illustrated in the Method. All attempts at replication are successful.
Randomization	Randomization was not applied for the study.
Blinding	Investigators are not blinded to group allocation during data collection and analysis.

Reporting for specific materials, systems and methods

We require information from authors about some types of materials, experimental systems and methods used in many studies. Here, indicate whether each material, system or method listed is relevant to your study. If you are not sure if a list item applies to your research, read the appropriate section before selecting a response.

Materials & experimental systems

n/a	Involved in the study	
☐	☒	Antibodies
☒	☐	Eukaryotic cell lines
☒	☐	Palaeontology and archaeology
☒	☐	Animals and other organisms
☐	☒	Human research participants
☒	☐	Clinical data
☒	☐	Dual use research of concern

Methods

n/a	Involved in the study	
☒	☐	ChIP-seq
☒	☐	Flow cytometry
☒	☐	MRI-based neuroimaging

Antibodies

Antibodies used	anti-LCN2; Proteintech, Cat.#26991-1-AP, Rabbit Polyclonal; dilution 1:100<br>anti-EPCAM; Proteintech, Cat.#66316-1-Ig, 2A2D5,Mouse Monoclonal; dilution 1:100<br>anti-Vimentin; Abcam, Cat.#ab92547,EPR3776, Rabbit monoclonal; dilution 1:200<br>anti-SAA1/2; Abmart, Cat.#TD6533S, Rabbit; dilution 1:100<br>anti-DKK4; Biorbyt, Cat.#orb449926, Rabbit polyclonal; dilution 1:100<br>anti-mouse Alexa Fluor 647; Abcam,Cat.#ab150115, Goat Anti-Mouse IgG H&L; Polyclonal;dilution 1:200<br>Cy3, Goat Anti-Rabbit IgG; abbkine,Cat.#A22220 , Goat Anti-Rabbit IgG;Polyclonal;dilution 1:200
Validation	All primary antibodies used in this study were validated by the manufacture.<br>anti-LCN2; Proteintech, http://www.ptgcn.com/products/LCN2-Antibody-26991-1-AP.htm; Pubmed ID:33352171<br>anti-EPCAM; Proteintech, http://www.ptgcn.com/products/EPCAM-Antibody-66316-1-Ig.htm<br>anti-Vimentin; Abcam, https://www.abcam.cn/vimentin-antibody-epr3776-cytoskeleton-marker-ab92547.html; Pubmed ID:33320843<br>anti-SAA1/2; Abmart, http://www.ab-mart.com.cn/page.aspx?node=65&id=30785<br>anti-DKK4; Biorbyt, https://www.biorbyt.com/dkk4-antibody-biotin-orb449926.html

Human research participants

Policy information about studies involving human research participants

Population characteristics	All of the women involved in the study were from Chinese Han nationality. The age of all subjects is among 44 to 55 years old. The past and current diagnosis of all subjects are consistent.
Recruitment	All individuals were not subjected to the chemotherapy before hysterectomy.<br>Endometrial lesions were found to be located at the fundus of uterine cavity.<br>No infectious diseases, such as hepatitis B, AIDS, syphilis, gonorrhea, were detected in any of the individuals.<br>Regular menstruation period previously, no family history of tumor.<br>All patients participated in the study with full informed consent. In order to narrow the bias, we selected patients with relatively consistent age and lesion location.
Ethics oversight	This study complied with all relevant ethical regulations and was approved by the Institutional Review Board (IRB) of the Obstetrics and Gynecology Hospital of Fudan University (No.2021-182).

Note that full information on the approval of the study protocol must also be provided in the manuscript.
